# Constituents and Anti-Hyperuricemia Mechanism of Traditional Chinese Herbal Formulae Erding Granule

**DOI:** 10.3390/molecules24183248

**Published:** 2019-09-06

**Authors:** Wugang Zhang, Wendi Du, Guofeng Li, Chen Zhang, Wuliang Yang, Shilin Yang, Yulin Feng, Haifang Chen

**Affiliations:** 1 National Pharmaceutical Engineering Center for Solid Preparation in Chinese Herb Medicine, Jiangxi University of Traditional Chinese Medicine, Nanchang 330004, China (W.Z.) (W.D.) (G.L.) (C.Z.) (S.Y.); 2 Key Laboratory of Modern Preparation of Traditional Chinese Medicine, Ministry of Education, Jiangxi University of Traditional Chinese Medicine, Nanchang 330004, China

**Keywords:** Erding granule, new indication, anti-hyperuricemia, active component analysis, pharmacological mechanism

## Abstract

Erding granule (EDG) is a traditional Chinese medicine that has recently been identified as having anti-hypouricemic effects. However, the active components and underlying mechanism for this new indication have not been elucidated. Therefore, we compared the effects of different EDG extracts (water, 50% ethanol and 95% ethanol) on serum uric acid concentrations in the hyperuricemia model mouse. We also analyzed the constituents of different extracts by ultra-high performance liquid chromatography combined with electrospray ionization quadrupole time-of-flight mass spectrometry (UHPLC-Q-TOF-MS/MS) to observe the variation between the active and inactive products. Extract activity and target site were evaluated by assessing uric acid- and inflammation-suppressing effects along with evaluating ability to regulate the uric acid transporter. The results showed that the 50% ethanol extract (EDG-50) had an obvious serum uric acid concentration lowering effect compared with water (EDG-S) and the 95% ethanol extract (EDG-95). UHPLC-Q-TOF-MS/MS analysis showed that EDG-50 was compositionally different to EDG-S and EDG-95. EDG-50 showed dose-dependent effects on reducing uric acid, suppressing inflammation and regulating uric acid transporters. Moreover, western blot analysis showed that EDG-50 down-regulated GLUT9 and URAT1 expression, and up-regulated OAT1 expression. Therefore, our findings enable the preliminarily conclusion that EDG-50 lowers serum uric acid concentrations, mainly by down-regulating the expression of GLUT9 and URAT1 proteins and up-regulating the expression of OAT1 proteins. This provides a research basis for clinical use of EDG as an anti-hyperuricemic agent.

## 1. Introduction

Hyperuricemia is human metabolic disorder resulting from increased uric acid formation and/or reduced uric acid elimination. Elevated serum uric acid concentrations may precipitate gout [1,2] and contribute to cardiovascular disease [3,4], type 2 diabetes mellitus [5], nonalcoholic fatty liver disease [6], and chronic kidney disease [7,8], which can seriously compromise human health. Reducing uric acid production with xanthine oxidase inhibitors and increasing uric acid excretion with diuretics are still the main treatments for lowering serum uric acid concentrations. However, with only allopurinol and febuxostat as the key drugs in clinical use, the availability of effective hypouricemic agents is very limited. The discovery of new uric acid-lowering drugs remains of considerable research interest [9,10,11,12].

Erding granule (EDG) is a prescription drug that could be found in the Chinese pharmacopoeia. It is composed of Viola yedoensis Makino, Taraxacum mongolicum Hand.-Mazz., Lobelia chinensi Lour, and Isatis indigotica Fort., and is boiled, concentrated, and granulated with sucrose or dextrin. EDG is often used to treat furuncles and sore throats due to its effect of clearing heat and dampness [13].

Previous studies showed that water extracts of EDG significantly reduced serum uric acid concentrations in the hyperuricemia mouse model, as well as suppressing abdominal capillary permeability induced by acetic acid, ear swelling incited by xylene, writhing caused by acetic acid in mice. EDG was also shown to alleviate swollen joints in rats with acute gouty arthritis induced by sodium urate crystal. The ultra-high performance liquid chromatography combined with electrospray ionization quadrupole time-of-flight mass spectrometry (UHPLC-Q-TOF-MS/MS) has been used to analyze the water extracts of EDG, with 198 components identified preliminarily. However, the active ingredients and mechanism for the hypouricemic effects of EDG remain unclear [14,15].

In this study, we compared the pharmacodynamic effects of different EDG polar extracts and then evaluated EDG using UHPLC-Q-TOF-MS/MS to identify the active constituents for comparison with other polar components to screen out the different parts. Concurrently, regulation of GLUT9, OAT1 and URAT1 uric acid transporter expression was detected by western blot analysis. This work offers an initial understanding of the active uric acid lowering constituents of EDG, and lays a foundation for further study of this new indication.

## 2. Results

### 2.1. Effects of EDG Extracts on Serum Uric Acid, Creatinine, and Blood Urea Nitrogen in the Hyperuricemia Mouse Model

The serum concentrations of uric acid, creatinine and blood urea nitrogen in mice with hyperuricemia were determined after intervention with different EDG extracts (EDG-S, EDG-50 and EDG-95). Table 1 shows that serum uric acid (SUA) (*P* < 0.01), creatinine (Cre) (*P* < 0.05), and blood urea nitrogen (BUN) (*P* < 0.05) concentrations were all significantly different in the model group (MG) compared with the control group (CG). Compared with the MG, UA and creatinine serum concentrations were decreased in all treatment groups, especially in the AP group (APG) (*P* < 0.01) and EDG-50 group (50G) (*P* < 0.05); creatinine and blood urea nitrogen concentrations in the APG were significantly increased (*P* < 0.01); EDG extracts with different polarity reduced creatinine and blood urea nitrogen, which was slightly significant in the 50G, but not significantly different in the MG. There was no significant change in the body weight of each mouse group over the 7-day course. Therefore, the 50% ethanol extract has a better anti-hyperuricemia effect than the water or 95% ethanol extract.

### 2.2. UHPLC-Q-TOF-MS/MS Analysis of EDG Extracts

**Figure 1:**
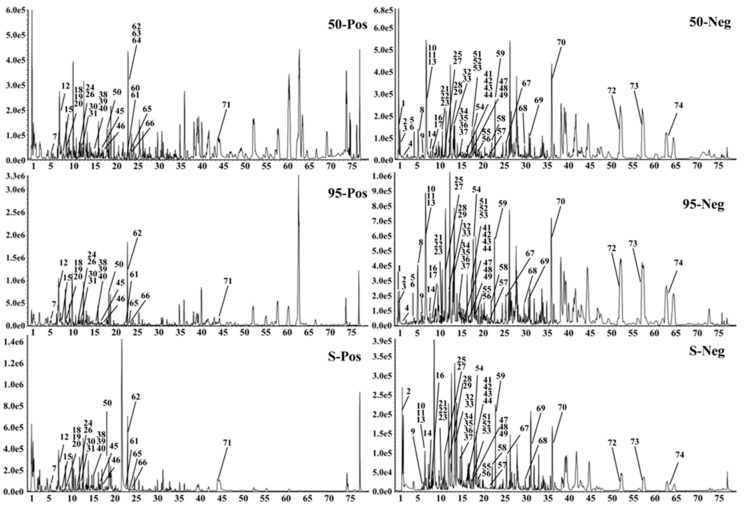
Base peak intensity chromatograms of the different extracts of EDG.

Comparison of the EDG-S, EDG-50 and EDG-95 preparations by UHPLC-Q-TOF-MS/MS analysis, revealed 74 components under positive and negative ion modes (positive ion mode: 23; negative ion mode: 51), with 72 being identified preliminarily (Figure 1 and Table 2). Of the 72 compounds, there were 21 organic acids, 9 coumarins, 28 flavone, 12 alkaloids, and 2 lignins. The structures of these compounds were tentatively assigned by matching the MS/MS data with a reference or public database such as PubChem (https://pubchem.ncbi.nlm.nih.gov/) or MassBank (http://www.massbank.jp/). By comparing the MS/MS intensity of the 74 components from the three extracts, we found that the response of the 20 components in EDG-50 was higher than that of EDG-S and EDG-95 (expressed as “Y” in Table 2).

### 2.3. Effects of EDG-50 on Serum Uric Acid, Creatinine, and Blood Urea Nitrogen in the Hyperuricemia Rat Model

We investigated the effects of EDG-50 on serum uric acid, creatinine and blood urea nitrogen concentrations in the hyperuricemia rat model. Table 3 shows that there was no significant change in rat body weight in the groups after 7 days of drug administration. The MG had significant differences in serum uric acid, creatinine, and blood urea nitrogen concentrations compared with the CG (*P* < 0.01). Compared with the MG, serum uric acid concentrations in the APG significantly decreased (*P* < 0.01), but there was no significant difference in creatinine or blood urea nitrogen concentrations. Compared with the MG, the different EDG-50 dose groups (EDG-50H, -50M and -50L) reduced serum uric acid and creatinine to varying degrees, which was most pronounced in the 50HG (*P* < 0.01). None of the EDG-50 groups had a significant effect on blood urea nitrogen.

### 2.4. Pathological Slices of EDG-50 on the Kidney of Hyperuricemic Rats

**Figure 2:**
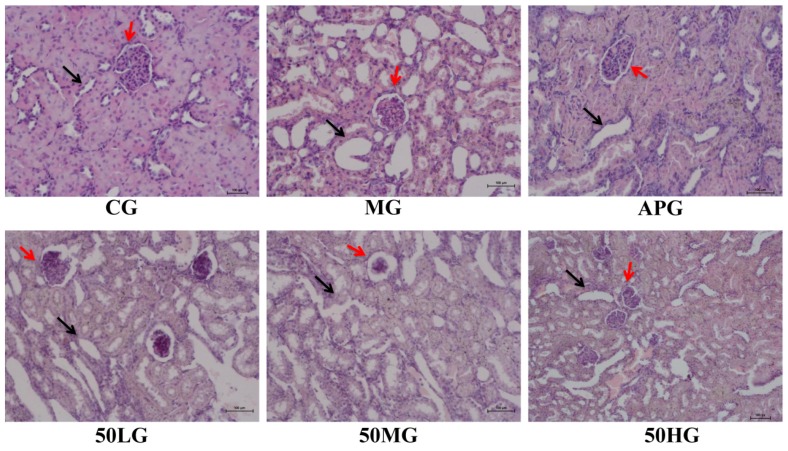
HE staining results (200×). Note: The black arrow points to the renal tubule, the red arrow points to the glomerulus.

Figure 2 shows that glomerular atrophy and renal tubular dilation were present in the MG, compared with the CG. Compared with MG, renal tubules in the APG were slightly dilated and the glomerular structure was clear; whereas the renal tubules and glomeruli of the 50HG and 50MG showed some improvement, but there was no significant improvement in the EDG-50LG.

### 2.5. Effect of EDG-50 on TNF-a, IL-1β and IL-6 in Kidney Tissue from Hyperuricemic Rats

Concentrations of the inflammatory factors (TNF-α, IL-1β and IL-6) in the renal tissues of hyperuricemic rats were measured after exposure to three different doses of EDG-50 (50H, 50M and 50L) (Table 4). Compared with the CG, the levels of the three inflammatory factors were all significantly increased in the MG (*P* < 0.01). Compared with the MG, TNF-α (*P* < 0.01), IL-1β (*P* < 0.05) and IL-6 (*P* < 0.01) were significantly decreased in the APG, while different doses of EDG-50 (50H, 50M and 50L) all decreased the three inflammatory factors with the effect most pronounced with 50HG.

### 2.6. Effect of EDG-50 on the Expression of OAT1, GLUT9, and URAT1 in Kidney Tissue from Hyperuricemic Rats

**Figure 3:**
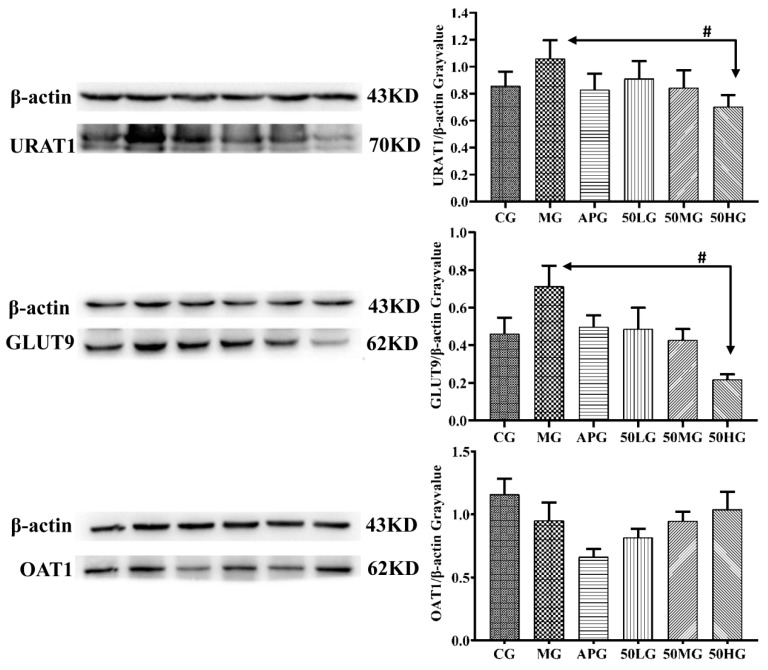
Effect of EDG-50 on the expression of OAT1, GLUT9, and URAT1.

Western blotting was used to detect the effects of different doses of EDG-50 (50H, 50M, and 50L) on the expression of uric acid excretion-related proteins (OAT1, GLUT9, and URAT1) in the kidney tissues of hyperuricemic rats. Figure 3 shows that the expression of GLUT9 and URAT1 was significantly up-regulated while the expression of OAT1 was significantly down-regulated in the C group after combined PO and HY administration. Compared with the MG, the expression of OAT1, GLUT9 and URAT1 protein in the AP group was down-regulated; whereas the expression of GLUT9 and URAT1 protein in the EDG-50 group was down-regulated, and the expression of OAT1 protein up-regulated in a dose-dependent manner; for the 50HG, the difference in down-regulated expression of GLUT9 and URAT1 protein was significant.

## 3. Discussion

Uric acid is the final product of human purine metabolism. Most uric acid in the human body is produced by the liver. Purine synthesis eventually leads to hypoxanthine, which is then metabolized by xanthine oxidase to uric acid. Therefore, Xanthine oxidase is the key factor to inhibit the production of uric acid [16,17]. In addition, uric acid excretion is mainly regulated by related uric acid transporters in the kidney, including fructose transporter (GLUT9), uric acid anion transporter 1 (URAT1), and organic anion transporters (OAT1, OAT3). URAT1 mediates the exchange of organic anions such as uric acid, lactic acid, chlorine, and other inorganic anions. Uric acid is reabsorbed into renal tubular epithelial cells from the tubular lumen. GLUT-9 mediates uric acid reabsorption across the basement membrane of tubular epithelial cells for return to the blood to help maintain equilibrium. OAT1 and OAT3 are expressed in the basolateral membrane of renal tubular epithelial cells and transport uric acid from peritubular capillaries to renal tubular epithelial cells to complete the first step of uric acid secretion [18,19,20,21]. These are the key ion channels that maintain uric acid concentrations in humans.

After we found that EDG has anti-hyperuricemic effects, we speculated that EDG might reduce serum uric acid concentration mainly via regulation of the uric acid transporter, and increase uric acid excretion. We confirmed that regulation of the uric acid transporter is in accordance with the findings for similar traditional Chinese medicines [22,23,24]. In addition, the previous experimental results were obtained with traditionally prepared EDG prescription drugs as a water extract. Therefore, in order to maximize the anti-hyperuricemic effect of EDG, the active fractions of EDG were screened. The experimental results showed that the anti-hyperuricemic effect of a 50% ethanol extract of EDG was better than that of traditional water and 95% ethanol extracts. Further, the anti-inflammatory effect of EDG-50 and the experimental results regarding regulation of the expression of uric acid transporter proteins showed a dose-response relationship. These results further revealed that a principle EDG effect is to lower serum uric acid concentrations. We concentrated on the 50% ethanol extract which down-regulated GLUT9 and URAT1 protein expression and up-regulated OAT1 protein expression. The results of UHPLC-Q-TOF-MS/MS analysis showed that the components of EDG-50 extracts were different from those of EDG-S and EDG-95 extracts. We inferred that the anti-hyperuricemic effect of EDG-50 was better than that of the EDG-S and EDG-95 extracts, mainly as a result of the different constituents, which preliminary results suggested amounted to 20 differences. These 20 different components include organic acids, alkaloids and flavonoids, which have been reported to have significant anti-hyperuricemia effects [25,26,27,28,29]. Therefore, it can be concluded that EDG produces anti-hyperuricemic effects by promoting uric acid excretion and improving renal function.

The above results only apply to the 50% ethanol extract of EDG, and the specific substances responsible for the anti-hyperuricemic actions are still unclear. Therefore, we will further separate and purify the 50% ethanol extract for future studies of EDG’s serum uric acid lowering and anti-inflammatory effects, and its regulation of uric acid transporter proteins. This will enable refined research on the pharmacodynamics of EDG prescription drugs.

## 4. Materials and Methods

### 4.1. Reagents

UHPLC grade acetonitrile and methanol were purchased from Fisher Scientific (Shanghai, China), and analytical grade formic acid from Sinopharm Chemical Reagent Co. Ltd. (Shanghai, China). Ultra-pure water was provided by Watsons (Hong Kong, China). The reference standards—gallic acid, esculetin, quinic acid, caffeic acid, 5-hydroxytryptophan, luteolin, and kempferol—had HPLC purity ≥ 98% and were purchased from Chengdu Must Bio-technology Co. Ltd. (Chengdu, China). Allopurinol (AP), hypoxanthine (HY), and potassium oxonate (OP) (purity ≥ 97%) were supplied by Sigma-Aldrich Corporation Co. Ltd. (St. Louis, MO, USA). The uric acid (UA) assay kit was obtained from Hitachi Ltd. (Tokyo, Japan), and the TNF-α, IL-1β, and IL-6 assay kits from the Neobioscience Technology Company (Shenzhen, China).

### 4.2. Plant Material and Preparation of the EDG Extracts

EDG prescription medicine (*Viola yedoensis Makino*, *Taraxacum mongolicum Hand.-Mazz.*, *Lobelia chinensi Lour.*, *Isatis indigotica Fort.*) was purchased from Jiangxi Zhangshu Tianqitang Traditional Chinese Medicine Pieces Co. Ltd. (Zhangshu, China) and stored in the National Engineering Research Center of Traditional Chinese Medicine Solid Preparation Manufacturing Technology, Jiangxi University of Traditional Chinese Medicine (Jiangxi, China).

Viola yedoensis Makino, Taraxacum mongolicum Hand.-Mazz., Lobelia chinensi Lour., Isatis indigotica Fort. were mixed in equal proportions (100 g per herb) to make three batches. These were extracted for one hour with water, 50% ethanol or 95% ethanol (at 10 times the herb volume), on two separate occasions. The extracts were then dried under reduced pressure generating 128.8 g of water extract (EDG-S), 96.76 g of 50% ethanol extract (EDG-50) and 40 g of 95% ethanol extract (EDG-95).

### 4.3. Experimental Animals

Male Sprague–Dawley (SD) rats (180–200 g) and male Kunming mice (18–22 g) were obtained from Hunan Slaike Jingda Laboratory Animal Co. Ltd. (Changsha, China). The animals were housed in plastic cages under standard laboratory conditions at 23 ± 1 °C, relative humidity 55% and 12 h light/dark cycle (lights on 7:00–19:00 h). All animal experiments were consistent with the Guide for the Care and Use of Laboratory Animals of Jiangxi University of Traditional Chinese Medicine (No. SYXK (Gai)-2017-0004).

### 4.4. Establishment of the Hyperuricemic Mouse Model and Experimental Protocol

Mice (n = 60) were divided randomly into six groups (n = 10 each): control group (CG), model group (MG), AP group (APG; 10 mg/kg), EDG-S group (SG, 5.8 g/kg/day, equivalent to 18 g raw materials/kg/day), EDG-50 group (50G, 4.4 g/kg/day, equivalent to 18 g raw materials/kg/day), and EDG-95 group (95G, 1.8 g/kg/day, equivalent to 18 g raw materials/kg/day). For the treatment groups, AP and EDG treatments were dispersed in 0.5% (*w/v*) sodium carboxymethyl cellulose (CMC-Na) and administered orally once daily at 8:00 AM from days 1 to 7 whereas the CG and MG received CMC-Na solution alone. After an overnight fast, hyperuricemia was established in all mouse groups except the CG using an intraperitoneal injection of the uricase inhibitor, PO (450 mg/kg), on day 7; the CG was treated with same volume of CMC-Na solution. After 1 h, all groups received their assigned treatments. Blood was collected 1 h after drug administration, and centrifuged at 4000 rpm at 4 °C for 10 min to separate the serum. Serum uric acid concentration was determined using the Hitachi 7080 automatic biochemical analyzer (Kyoto, Japan).

### 4.5. Establishment of the Hyperuricemic Rat Model and Experimental Protocol

After acclimatizing 60 rats for one week, they were divided randomly into the following six groups (n = 10 each): control group (CG), model group (MG), AP group (APG; 10 mg/kg), EDG-50 high dose group (50HG, 2.9 g/kg/day, equivalent to 12 g raw materials/kg/day), EDG-50 medium dose group (50MG, 1.5 g/kg/day, equivalent to 6 g raw materials/kg/day), and EDG-50 low dose group (50LG, 0.8 g/kg/day, equivalent to 3 g raw materials/kg/day). During each of the 7 experimental days, HY 300 mg/kg was administered to all except the CG rats by oral gavage followed by intraperitoneal injection of OP 200 mg/kg at 0800 h each day to induce hyperuricemia. One hour later, the group-specific solvent/drug was given by oral gavage. Blood was collected from the rats 1 h after drug administration, and centrifuged at 4000 rpm at 4 °C for 10 min to separate the serum. After sacrificing the animals, the kidneys were collected and flash frozen in liquid nitrogen. All samples were stored at -80 °C until analysis. Serum uric acid concentrations were measured by the Hitachi 7080 automatic biochemical analyzer (Kyoto, Japan).

### 4.6. UHPLC-Q-TOF-MS/MS Analysis

UHPLC analyses were carried out on a Shimadzu system (Kyoto, Japan) equipped with a LC-3AD solvent delivery system, SIL-30ACXR auto-sampler, CTO-30AC column oven, DGU-20A3 degasser, and CBM-20A controller. The analytical column was a Waters Acquity UPLC BEH C_18_ column (100 mm × 2.1 mm, 1.7 um). The column oven temperature was set at 40 °C. The mobile phases consisted of water containing 0.1% formic acid (solvent A) and acetonitrile (solvent B). The flow rate was set at 0.3 mL/min. The binary gradient was applied with linear interpolation as follows: 0.01 min, 5% B; 2.0 min, 5% B; 18.0 min, 20% B; 40 min, 55% B; 50 min, 65% B; 60 min; 70% B; 73 min, 80% B; 74 min, 100% B; 76 min, 100% B; and 77 min, 5% B.

The UHPLC-Q-TOF-MS/MS detection was conducted on a Triple TOFTM 5600+ system with a Duo Spray source in the negative electrospray ion mode (AB SCIEX, Foster, CA, USA). The electrospray ionization was applied in the negative mode with the following parameters: ion spray voltage, −4500 V; ion source temperature, 500 °C; curtain gas, 25 psi; nebulizer gas (GS 1), 50 psi; heater gas (GS 2), 50 psi; and declustering potential (DP), −100 V. The mass ranges were set at m/z 50–1250 Da for the TOF-MS scan and 50–1250 Da for the TOF MS/MS experiments. In the IDA-MS/MS experiment, the collision energy (CE) was set at 35 eV, and the collision energy spread (CES) was (±) 15 eV for the UHPLC-Q-TOF-MS/MS detection. Accurate mass and composition for the precursor and fragment ions were analyzed using Peak View^®^ 1.2 software (AB SCIEX, Foster City, CA, USA)

### 4.7. Pathological Section Analysis

The collected right rat kidney was fixed in 4% paraformaldehyde solution for 24 h, and then cut into appropriate thicknesses, rinsed with running water for 2 h, dehydrated, embedded and sectioned. HE staining was used to observe the pathological damage under microscope.

### 4.8. Kidney Cytokine Analysis

Concentrations of three inflammatory factors (TNF-α, IL-1β and IL-6) were measured in tissues using ELISA kits, as per the manufacturer’s instructions, at 450 nm absorbance. Inflammatory factor concentrations were calculated according to the formula of the drawn standard curves.

### 4.9. Western Blot Analysis of Kidney Tissues

Renal tissue was homogenized with RIPA buffer and centrifuged at 14,000 rpm and 4 °C for 30 min to obtain total protein. Protein concentration was then established by BCA Protein assay kit (Beyotime, Shanghai, China). Equal quantities of tissue lysate (30 µg protein) were separated by 8% SDS-PAGE, followed by western blot analysis after transfer of the separated proteins to PVDF membranes. After incubation in a 5% nonfat milk blocking suspension for 1 h, the membranes were incubated with antibodies against β-actin, URAT1, GLUT9, and OAT1 at 4 °C overnight. HRP-conjugated secondary antibodies were incubated at a 1:5000 dilution for 2 h with the membranes. The detection was done using a Molecular Imager ChemiDoc XRS + System (Bio-Rad, Hercules, CA, USA).

### 4.10. Statistical Analysis

Data are presented as the mean ± standard error (S.E.) and were statistically evaluated using one-way analysis of variance (ANOVA) with the GraphPad Prism (GraphPad Inc., San Diego, CA, USA) software program. The results were considered statistically significant at *P* < 0.05.

## 5. Conclusions

Our results showed that EDG-50 was more effective at lowering serum uric acid concentrations than EDG-S or EDG-95. Therefore, our pharmacodynamic evaluation focused mainly on the 50% ethanol extract. We found that EDG-50 had a dose-dependent relationship with anti-hyperuricemia and anti-inflammatory effects, as well as with the regulation of uric acid transporter protein expression. The analysis of EDG-S, EDG-50, and EDG-95 by UHPLC-Q-TOF-MS/MS detected 74 components under positive and negative ion modes, with 20 components of EDG-50 responding better than EDG-S and EDG-95. In addition, western blotting results showed that EDG-50 inhibited hyperuricemia mainly by down-regulating GLUT9 and URAT1 protein expression and up-regulating OAT1 protein expression.

## Figures and Tables

**Table 1:** Effects of the different extracts of Erding granule (EDG) on hyperuricemia model mice (n = 10).

Group	Dose (g/kg)	Weight	SUA (µmol/L)	Cre (µmol/L)	BUN (mg/dL)
CG	−	31.75 ± 1.24	104.72 ± 32.61	5.98 ± 1.41	15.96 ± 1.74
MG	0.45	30.32 ± 1.64	176.34 ± 53.63 **	6.89 ± 0.65 *	17.30 ± 3.37 *
APG	0.015	27.31 ± 4.56	47.60 ± 24.07 ##	10.79 ± 4.51 ##	25.15 ± 7.66 ##
SG	7.73	30.09 ± 2.50	142.30 ± 48.90	6.53 ± 1.09	16.74 ± 2.23
50G	5.81	31.09 ± 2.16	121.98 ± 40.87 #	5.42 ± 1.25 #	15.12 ± 4.38
95G	2.40	31.09 ± 1.89	138.04 ± 18.98	6.62 ± 3.47	15.03 ± 3.93

Note: “*” was compared with the blank group, * *P* < 0.05, **.P < 0.01; “#” was compared with the model group, # *P* < 0.05, ## *P* < 0.01. CG—control group; MG—model group; APG—AP group; SG—EDG-S group; 50G—EDG-50 group; 95G—EDG-95 group.

**Table 2:** Identification of the chemical constituents in the different extracts of EDG by ultra-high performance liquid chromatography combined with electrospray ionization quadrupole time-of-flight mass spectrometry (UHPLC-Q-TOF-MS/MS).

No.	Formula	Mass /Da	Adduct	Found At Mass/Da	Main MS/MS Productions	EDG-50			EDG-95			EDG-S			Proposed Compound	
						Error /ppm	RT /min	Intensity	Error /ppm	RT /min	Intensity	Error /ppm	RT /min	Intensity		
1	C_43_H_32_N_2_O_9_	720.2108	−H	719.2035	719.2004, 377.0833, 341.1069, 215.0317, 179.0558, 161.0465	−4.5	0.9	224,431	−0.8	0.9	11,042	/	/	/	Chelidimerine	Y
2	C_6_H_8_O_7_	192.0270	−H	191.0197	129.0213, 111.0108, 87.0127	4	1.2	250,963	2.9	1.2	87,211	−9.2	1.22	822,532	Citric acid	
3	C_10_H_13_N_5_O_5_	283.0917	−H	282.0844	150.0426, 133.0172, 108.0221	0	1.3	39,988	1.5	1.31	10,689	/	/	/	Isoguanosine	Y
4	C_7_H_6_O_5_	170.0215	−H	169.0143	125.0278, 79.0227	6.5	1.61	12,942	2.3	1.63	18,513	/	/	/	Gallic*	
5	C_13_H_12_O_9_	312.0481	−H	311.0409	179.0352, 161.0228, 149.0098, 135.0465, 117.0372, 107.0526	0	4.35	63,159	−1	4.35	63,500	/	/	/	Caftaric acid	
6	C_16_H_18_O_9_	354.0951	−H	353.0878	191.0561, 179.0359, 135.0466	−0.9	4.37	18,618	−2.1	4.38	24,046	/	/	/	Neochlorogenic acid	
7	C_9_H_6_O_4_	178.0266	+H	179.0339	179.0341, 133.0285, 123.0449, 105.0350	−0.9	5.41	109,346	−0.6	5.46	322,194	−2.4	5.25	99,156	Esculetin*	
8	C_15_H_16_O_9_	340.0794	−H	339.0722	177.0201, 149.0249, 133.0309	−1	5.48	563,845	−0.4	5.49	1,624,129	/	/	/	Escosyl	
9	C_13_H_12_O_8_	296.0532	−H	295.0459	179.0318, 133.0157, 115.0064	0.8	6.53	6076	4	6.52	5721	6.4	6.61	410	Caffeoylmalic acid	Y
10	C_7_H_12_O_6_	192.0634	−H	193.0707	191.0779, 173.0513, 127.0392, 109.0298	4.4	7.11	52,340	4.4	7.09	99,317	2.3	7.12	39,686	Quinic acid*	
11	C_16_H_18_O_9_	354.0951	−H	353.0878	191.0574, 173.0471, 161.0256, 135.0466	−0.3	7.12	145,276	−0.7	7.09	269,510	−2.6	7.12	97,406	Chlorogenic Acid	
12	C_9_H_6_O_4_	178.0266	+H	179.0339	179.0336,133.0285,123.0444, 105.0341	−0.1	7.34	1,045,590	−0.1	7.33	2047,773	0	7.3	1,021,466	Isoesculetin	
13	C_9_H_8_O_4_	180.0423	−H	179.0350	179.0366, 163.0400, 135.0469, 117.0357, 107.0528	4.2	7.76	145,065	5.6	7.75	128,114	0.4	7.77	1852	Caffeicacid*	Y
14	C_16_H_18_O_9_	354.0951	−H	353.0878	191.0566, 179.0366,173.0461, 135.0466	−1	8.08	46,077	−0.8	8.1	62,346	−0.6	8.06	492	Cryptochlorogenic acid	
15	C_24_H_39_NO_8_	469.2676	+H	470.2748	470.2723, 308.2226, 220.1328, 174.1291	−0.9	8.16	4880	−11.1	8.08	1256	−3.1	8.09	1824	N-lauryl glucoside	Y
16	C_10_H_12_O_5_	212.0685	−H	211.0612	196.0376, 177.0206, 152.0490, 137.0258, 109.0310	2.1	8.28	53,287	3.3	8.29	97,264	−8.6	8.22	288	Gallic acid propyl ester	
17	C_10_H_8_O_4_	192.0423	−H	191.0350	191.0377, 176.0120, 161.0247, 148.0172, 135.0465	4.3	8.82	21,352	4.5	8.84	49,495	/	/	/	Methylesculetin	
18	C_24_H_39_NO_8_	469.2676	+H	470.2748	470.2761, 308.2204, 290.2122, 220.1346, 174.1286	−4.1	8.88	5605	−0.7	8.9	5048	0.5	8.81	1833	N-lauryl glucoside	Y
19	C_24_H_39_NO_8_	469.2676	+H	470.2748	470.2709, 308.2218, 220.1361, 174.1282	−4.1	9.02	4992	−2.5	9.02	3306	0.2	8.96	2417	N-lauryl glucoside	Y
20	C_11_H_12_N_2_O_3_	220.0848	+H	221.0921	176.0708, 158.0601, 147.0330, 133.0521, 104.0522	−2.9	9.42	8886	−0.9	9.41	5539	−0.8	9.37	12,999	5-Hydroxytryptophan*	
21	C_32_H_44_O_16_	684.2629	−H	683.2557	683.2691, 521.2005, 359.1449, 329.1396, 192.0793	−0.8	10.87	45,283	−0.1	10.97	69,562	−4.5	11.29	15,162	Clemastanin B	
22	C_33_H_40_O_20_	756.2113	−H	755.2040	755.2034, 609.1450, 430.0890, 284.0320	0	10.91	35,697	0.5	11.03	97,735	−2.7	11.3	20,076	Manghaslin	
23	C_27_H_30_O_15_	594.1585	−H	593.1512	593.1487, 503.1170, 473.1066, 383.0757, 353.0656, 325.0712, 297.0765	−0.9	11.13	591,640	−0.8	11.22	1,735,954	−3.9	11.05	5282	Vicenin-2	
24	C_24_H_39_NO_8_	469.2676	+H	470.2748	470.2721, 308.2228, 290.2101, 220.1354, 174.1265	−3.6	11.15	8351	−3.8	11.15	5398	−3.3	11.1	2297	N-lauryl glucoside	Y
25	C_9_H_10_O_5_	198.0528	−H	197.0456	197.0447, 169.0157, 124.0182	1.9	11.46	12,433	4.4	11.49	12,136	0	11.49	31	Gallic acid ethyl	Y
26	C_24_H_39_NO_8_	469.2676	+H	470.2748	470.2734, 308.2224, 220.1331, 174.1299	−4.2	11.58	8704	−3	11.59	5541	−1.8	11.54	2927	N-lauryl glucoside	Y
27	C_9_H_6_O_3_	162.0317	−H	161.0244	161.0257, 133.0311, 117.0385, 105.0366	5.7	11.68	9975	6.8	11.7	27,807	20.5	11.58	5314	Hydroxycoumarin	
28	C_27_H_26_O_18_	638.1119	−H	637.1046	637.1003, 351.0553, 285.0397, 193.0350	−2.2	12.18	45,258	−0.1	12.19	30,835	−6.7	12.18	815	Luteolin-O-glucuronosyl-glucuronide	Y
29	C_10_H_8_O_4_	192.0423	−H	191.0350	176.0124, 148.0183, 120.0248, 104.0305	4.9	12.53	81,183	5.1	12.53	327,187	0.2	12.42	1873	Methylesculetin	
30	C_21_H_20_O_11_	448.1006	+H	449.1078	431.0973, 413.0860, 395.0753, 329.0654, 299.0551, 165.0181, 137.0237	−1.6	12.84	231,975	−2.7	12.9	682,055	−0.9	12.85	389,268	Orientine	
31	C_21_H_20_O_11_	448.1006	+H	449.1078	449.1103, 431.0971, 413.0853, 329.0664, 299.0552, 137.0242	−0.9	13.23	41,259	−4.4	13.28	171,088	−1.1	13.25	72,452	Isoorientin	
32	C_13_H_12_O_9_	312.0481	−H	311.0409	193.0464, 179.0363, 149.0113, 135.0469, 117.0364, 107.0364	−0.7	13.68	59,343	0.2	13.75	409	−1.5	13.92	518	Caftaric acid	Y
33	C_22_H_18_O_12_	474.0798	−H	473.0726	311.0396, 293.0281, 219.0292, 191.0343, 179.0354, 161.0252, 149.0101	−2.5	13.69	408,502	−1.7	13.75	105,979	−3.6	13.93	1,385,318	Chicoric acid	
34	C_20_H_18_O_11_	434.0849	−H	433.0776	433.0750, 301.0352, 300.0276, 151.0043	−1	14.93	76,758	−1.4	14.93	198,601	−3	15.08	92,618	Quercetin-O-pentose	
35	C_21_H_20_O_10_	432.1057	−H	431.0984	431.0960, 353.0656, 323.0548, 311.0550, 293.0460, 283.0607, 269.0455	−1.6	15.02	68,567	−1.7	15.02	144,516	−3.8	15.13	15,145	Vitexin	
36	C_27_H_30_O_14_	578.1636	−H	577.1563	577.1537, 457.1095, 413.0858, 355.0767, 341.0666, 323.0555, 311.0522, 293.0451, 281.0456, 269.0450	−0.6	15.12	8253	−0.1	15.12	23,760	−3.4	15.21	4334	Vitexin-O-rhamnoside	
37	C_27_H_30_O_14_	578.1636	−H	577.1563	577.1533, 487.1188, 473.1075, 457.1112, 413.0866, 395.0777, 383.0768, 365.0655, 353.0658, 323.0545, 297.0796, 163.0373	−1.1	15.38	10,624	−0.7	15.38	47,674	−3.5	15.44	5620	Violanthin	
38	C_21_H_20_O_11_	448.1006	+H	449.1078	449.2466, 287.0562, 269.0484	−0.6	15.6	13,347	−0.2	15.56	19,278	−0.1	15.56	12,257	Kaempferol-O-glucoside	
39	C_27_H_30_O_15_	594.1585	+H	595.1658	595.2385, 449.1088, 287.0562, 153.0216	−1.2	15.65	55,584	−4.3	15.61	199,283	0.2	15.62	73,538	Nicotiflorine	
40	C_21_H_20_O_11_	448.1006	+H	449.1078	287.0555, 153.0187	−2	15.87	102,110	−3.2	15.86	319,417	−0.3	15.87	127,967	Trifolin	
41	C_22_H_22_O_11_	462.1162	−H	461.1089	461.1077, 371.0757, 353.0665, 341.0656, 313.0707, 298.0476, 269.0462	−1.7	16.04	72,756	−1.4	16.03	199,519	−5.5	16.11	48,781	Scoparin	
42	C_27_H_30_O_14_	578.1636	−H	577.1563	577.1551, 431.0975, 285.0398, 255.0300	−0.2	16.55	76,563	−1.1	16.54	148,515	−4.4	16.46	32,802	Kaempferitrin	
43	C_20_H_18_O_11_	434.0849	−H	433.0776	433.0757, 301.0342, 300.0267, 151.0042	−1.9	16.79	42,967	−1.1	16.79	108,180	−5	16.71	49,014	Quercetin-O-pentose	
44	C_10_H_8_O_4_	192.0423	−H	191.0350	191.0444, 176.0110, 148.0173, 120.0256	3.9	16.83	14,520	2.3	16.84	19,384	16.8	16.82	129	Methylesculetin	
45	C_11_H_10_O_5_	222.0528	+H	223.0601	223.0617, 207.0304, 190.0266, 162.0319, 134.0366	−0.3	17.07	96,677	−1.1	17.12	204,839	1.1	17.1	78953	Hydroxy-dimethoxy-cumarin	
46	C_21_H_20_O_11_	448.1006	+H	449.1078	449.2300, 287.0548,	−1.4	17.51	10,684	−3.7	17.61	34,955	0.3	17.6	17,749	Astragalin	
47	C_34_H_42_O_20_	770.2269	−H	769.2197	769.2176, 299.0559, 284.0315	0.5	17.81	18,121	0.7	17.81	40,329	−3	17.72	9244	Kaempferide-O-Diglucoside-O-pentose	
48	C_27_H_30_O_14_	578.1636	−H	577.1563	577.1547, 269.0452	−1.4	17.83	13,543	0	17.82	58,263	−3.8	17.77	11,331	Rhoifolin	
49	C_16_H_10_O_8_	330.0376	−H	329.0303	329.0316, 311.0205, 285.0401, 267.0291, 243.0302	0	17.86	30,787	−1	18.04	51,352	−1.7	17.84	27,116	Dimethoxy ellagic acid	
50	C_11_H_10_O_5_	222.0528	+H	223.0601	223.0619, 208.0376, 193.0140, 165.0188, 147.0086	−0.5	18.29	70,026	−1.7	18.35	183,868	0.1	18.34	56,086	Hydroxy−dimethoxy-cumarin	
51	C_28_H_36_O_13_	580.2156	−H	579.2083	417.1532, 402.1299, 387.1060, 181.0510, 166.0275, 151.0051, 137.0251	−1.6	18.3	75,525	−0.6	18.29	146,736	−2.8	18.26	34,715	Syringaresinol-O-glucopyranoside	
52	C_21_H_20_O_10_	432.1057	−H	431.0984	431.0972, 268.0366	−2.6	18.36	14,511	−1.7	18.36	36,311	−2.5	18.35	8892	Apigenin-7-O-glucoside	
53	C_27_H_32_O_14_	580.1792	−H	579.1719	579.1689, 459.1130, 313.0709, 271.0608, 193.0146, 177.0182, 151.0042	−0.8	18.47	51,833	−1.2	18.44	28,001	−4.2	18.44	11,215	Naringin	Y
54	C_34_H_42_O_20_	770.2269	−H	769.2197	769.2171, 623.1965, 299.0547, 284.0312	−1	18.76	6943	−0.5	18.76	21,178	−1.8	18.7	5930	Kaempferide-O-Diglucoside-O-pentose	
55	C_28_H_32_O_15_	608.1741	−H	607.1668	607.1651, 299.0557, 284.0324	−0.3	19.03	584,894	0.2	19.02	2,556,400	−3.7	18.98	692,927	Diosmine	
56	C_25_H_24_O_12_	516.1268	−H	515.1195	515.1211, 353.0869,191.0562, 179.0355, 173.0464, 135.0461	−0.9	19.48	217,297	−1.7	19.48	189,989	−3.4	19.43	168,824	Isochlorogenicacid B	Y
57	C_16_H_10_O_8_	330.0376	−H	329.0303	285.0424, 257.0451, 242.0204	−0.4	22.04	10,197	−0.6	22.04	35,556	−1.3	22.03	3323	Dimethoxy ellagic acid	
58	C_15_H_10_O_6_	286.0477	−H	285.0405	285.0400, 217.0509, 199.0410, 151.0051, 133.0311	0.7	22.69	332,783	1.4	22.69	689,966	0.8	22.69	264,998	Luteolin*	
59	C_11_H_12_O_4_	208.0736	−H	207.0663	207.0663, 179.0361, 161.0249, 133.0306	3.1	23.17	129,947	4.3	23.16	73,165	4.9	23.22	228	Ethyl caffeate	Y
60	C_27_H_43_NO_2_	413.3294	+H	414.3367	414.3378, 396.3251, 271.2068, 253.1958, 197.1327, 157.1011	−1.7	23.3	17,266	/	/	/	/	/	/	Solasodine	Y
61	C_28_H_32_O_14_	592.1792	+H	593.1865	593.1877, 447.1279, 285.0752, 242.0561, 153.0173	−0.6	23.43	1,878,046	−4.8	23.45	7,898,989	0.1	23.43	3,084,436	Acaciin	
62	C_21_H_20_O_10_	432.1057	−H	431.0984	431.0963, 285.0393, 284.0318, 257.0447, 151.0046	0	23.5	16,828	0	23.5	58,554	−4.8	23.48	6075	Kaempferol-3-Rhamnoside	
63	C_27_H_43_NO_2_	413.3294	+H	414.3367	414.3383, 396.3242, 271.2069, 253.1966, 171.1188, 157.1023	−1	23.57	34,218	/	/	/	/	/	/	Solasodine	Y
64	C_27_H_43_NO_2_	413.3294	+H	414.3367	414.3366, 396.3234, 271.2052, 253.1944, 197.1313, 157.1025	−2.1	23.77	10,032	/	/	/	/	/	/	Solasodine	Y
65	C_28_H_34_O_14_	594.1949	+H	595.2021	595.3527, 329.1011, 287.0923, 153.1084	−2.7	23.83	42,120	−5.1	23.86	68,113	−1.1	23.84	40,009	Isosakuranetin−O-rutinoside	
66	C_27_H_45_NO_2_	415.3450	+H	416.3523	416.3537, 398.3403, 273.2196, 255.2115, 161.1323	−3	24.12	10,349	4.4	24.13	611	0.4	24.18	434	Tomatidine	Y
67	C_15_H_10_O_6_	286.0477	−H	285.0405	285.0388, 229.0524, 211.0391, 183.0470, 143.0472	0.1	25.73	10,541	0.4	25.74	33,472	−0.5	25.74	10,143	Kempferol*	
68	C_11_H_14_O_3_	194.0943	−H	193.0870	193.0870, 177.0553, 163.0360	1.3	30.08	10,787	4.4	30.08	8480	3.3	30.09	9162	Butylparaben	Y
69	C_16_H_12_O_5_	284.0685	−H	283.0612	283.0612, 268.0376, 239.0347, 211.0399, 151.0042, 1170364	2.4	31.73	411,240	1.9	31.73	966,434	2.1	31.76	821,365	Acacetin	
70	C_30_H_48_O_5_	488.3502	−H	487.3429	487.3407, 443.3155, 425.3410, 391.2990, 319.2477, 207.1753	−1.1	36.75	1,790,552	−1	36.74	2,880,239	−2.9	36.79	709,844	Asiatic Acid	
71	C_12_H_14_O_4_	222.0892	+H	223.0965	207.0336, 191.0012, 162.9689, 149.0244, 133.0138, 121.0304	−1.2	44.56	19,655	−1.6	44.52	20,718	−2.1	44.52	20,676	Ferulic acid ethyl ester	
72	C_30_H_48_O_3_	456.3604	−H	455.3531	455.3511, 407.3290	−0.5	52.64	175,825	−1.2	52.77	451,419	−3.7	52.61	21,661	N	
73	C_30_H_46_O_3_	454.3447	−H	453.3374	453.3367, 407.3354	−0.6	57.83	18,035	−2.4	57.82	56,886	−3.7	57.83	2653	N	
74	C_16_H_32_O_2_	256.2402	−H	255.2330	255.2328, 237.2328, 214.9932	1.4	63.62	486,504	5.8	63.63	1,034,687	3.4	63.57	88,067	Palmitic acid	

Note: “*”: The components were unambiguously identified by comparison with the reference standards. “N”: Not identified. “Y”: The response of these compounds in EDG-50 is higher than that in EDG-95 and EDG-S.

**Table 3:** Effects of the EDG-50 on hyperuricemia model rat (n = 10).

Group	Doseg/(kg)	Weight (g)	SUA (µmol/L)	Cre (µmol/L)	BUN (mg/dL)
CG	−	272.70 ± 16.81	74.38 ± 14.64	20.22 ± 2.16	13.40 ± 2.81
MG	−	261.80 ± 19.77	499.21 ± 101.11 **	32.07 ± 9.44 **	21.21 ± 5.77 **
APG	0.01	250.60 ± 23.62	139.23 ± 26.19 ^##^	32.13 ± 5.49	22.98 ± 5.10
50HG	3.84	269.70 ± 10.76	393.89 ± 167.72 ^#^	24.76 ± 2.91 ^##^	17.34 ± 3.46
50MG	1.92	266.40 ± 13.41	414.12 ± 178.26	31.64 ± 6.79	24.89 ± 5.27
50LG	0.96	258.90 ± 17.38	437.92 ± 127.00	28.69 ± 5.58	26.11 ± 10.21

Note: “*” was compared with the blank group, * *P* < 0.05, ** *P* < 0.01; “#” was compared with the model group, # *P* < 0.05, ## *P* < 0.01.

**Table 4:** Effect of EDG-50 on inflammatory factors in kidney tissue of HUA model rats (n = 10).

Group	TNF-α	IL-1β	IL-6
CG	9.72 ± 0.67	2.47 ± 0.56	2.81 ± 0.05
MG	14.38 ± 2.24 **	4.20 ± 0.82 **	4.45 ± 0.56 **
APG	10.74 ± 2.62 ^##^	3.22 ± 0.80 ^#^	3.26 ± 0.51 ^##^
50HG	9.50 ± 1.04 ^##^	3.25 ± 0.72 ^#^	2.57 ± 0.47 ^##^
50MG	11.59 ± 3.34	3.83 ± 0.57	3.57 ± 0.44 ^##^
50LG	13.98 ± 1.90	4.08 ± 0.79	3.91 ± 0.17 ^#^

Note: “*” was compared with the blank group, * *P* < 0.05, ** *P* < 0.01; “#” was compared with the model group, # *P* < 0.05, ## *P* < 0.01.
